# Health literacy related to climate change: a communıty-based cross-sectional study

**DOI:** 10.3389/fpubh.2026.1779499

**Published:** 2026-05-15

**Authors:** Emine Ekici, M. Akif Sezerol, Ozan Ozkol

**Affiliations:** 1 School of Nursing, Maltepe University, Istanbul, Türkiye; 2 Department of Public Health, Hamidiye Faculty of Medicine, University of Health Sciences, Istanbul, Türkiye; 3 Epidemiology Program, Institute of Health Sciences, Istanbul Medipol University, Istanbul, Türkiye

**Keywords:** climate change, community-based study, health literacy, public health, vulnerable populations

## Abstract

**Introduction:**

Climate change poses health risks to populations, particularly in socioeconomically disadvantaged urban communities with limited adaptive capacity. However, evidence on climate change–related health literacy, defined as individuals’ ability to access, understand, and use information on climate-related health risks, remains limited. This study aimed to assess climate change–related health literacy among adults living in a disadvantaged district of Istanbul and to examine its association with selected demographic characteristics related to vulnerability and adaptation.

**Methods:**

A community-based cross-sectional study was conducted between July and September 2025 among adults aged 18–65 years registered with family physicians in Sultanbeyli, Istanbul. Using simple random sampling, 1,182 individuals were contacted, and data from 441 participants were analyzed. Data were collected using a demographic information form and the 24-item Climate Change Health Literacy Scale. Independent samples *t*-tests and one-way ANOVA were used for analysis.

**Results:**

The mean age of participants was 38.7 ± 12.1 years, and 53.3% were female. Most participants (94.6%) had not received formal education on climate change, and social media was the main source of information (65.1%). Only 21.8% reported sufficient knowledge of climate change–related health impacts. The mean total health literacy score was 94.4 ± 14.0, indicating a moderate level of literacy. Higher scores were observed among women, individuals with higher education, and those who regularly followed climate-related news (*p* < 0.05). No significant differences were found by income or occupation.

**Discussion:**

These findings reveal gaps between knowledge and the adoption of protective behaviors in disadvantaged urban communities. Community-based health education initiatives are essential to strengthen adaptive capacity.

## Background

Climate change represents one of the most pressing global challenges, with profound impacts on human health. Increasing extreme weather events, air pollution, disrupted food and water security, and shifts in infectious disease patterns pose significant risks and strain health systems (1–4). In Türkiye, the climate crisis has led to severe droughts, wildfires, flash floods, diminishing water resources, and agricultural losses, with regional variations such as increasing wildfires in the Mediterranean and accelerating desertification in Central and Southeastern Anatolia. Irregular rainfall further contributes to more frequent and severe floods (5). In the Marmara Region, particularly in Istanbul, climate change is associated with extreme temperatures, flood risks, water scarcity, and rising sea levels, all exacerbated by high population density and unplanned urbanization (6).

These impacts are not evenly distributed, as socioeconomically disadvantaged urban communities are more exposed and have reduced capacity to adapt due to limited resources and restricted access to reliable health information (3, 7, 8). Strengthening community resilience and adaptive responses has therefore become a public health priority (9).

Health literacy plays a key role in this process, as it refers to individuals’ ability to access, understand, critically evaluate, and apply health-related information, thereby influencing risk perception and the adoption of protective behaviors (9, 10). In the context of climate change, health literacy extends beyond general health knowledge to include awareness of climate-related health risks, understanding of early warning information, and engagement in adaptive behaviors (11, 12). Accordingly, it contributes to adaptive capacity by enabling individuals to respond appropriately to climate-related health risks, such as taking protective measures during extreme heat events. Evidence shows that individuals with higher health literacy are more likely to engage in health-protective behaviors, seek reliable information, and support collective health actions (13). Conversely, limited health literacy may deepen existing inequalities by reducing individuals’ ability to respond effectively to climate-related risks. This is particularly important for disadvantaged urban populations with higher exposure and lower access to accurate information (11). Although climate change awareness is increasing, a gap remains between knowledge and its translation into health-related action. Health professionals, especially nurses, are well positioned to address this gap through their roles as educators and community advocates in primary care and public health settings (11, 14, 15). However, empirical evidence on climate change–related health literacy at the community level remains limited, particularly in disadvantaged urban contexts, where most studies have focused on awareness rather than behavioral adaptation (16, 17).

In Türkiye, although public awareness of climate change is relatively high, climate-related health literacy remains limited, particularly regarding health impacts and protective behaviors (18). National data suggest that this limitation is more pronounced among individuals with lower socioeconomic and educational status (19). Sultanbeyli, a socioeconomically disadvantaged district of Istanbul characterized by rapid urbanization, migration, and vulnerable populations, provides an important setting to examine these issues. This study aimed to investigate the level of climate change–related health literacy and its association with demographic variables in this context.

## Methods

### Study design and setting

This study was conducted as a community-based cross-sectional study in Sultanbeyli, a socioeconomically disadvantaged district of Istanbul. Sultanbeyli has a population of 369,193, approximately 6% of whom are refugees, and is characterized by rapid urbanization, high internal migration, and a concentration of vulnerable populations (20–22). In Türkiye, all individuals are registered with a family physician; therefore, the study was conducted between July and September 2025 among adults aged 18–65 years registered with family physicians in Sultanbeyli.

### Population and sampling

**Figure 1:**
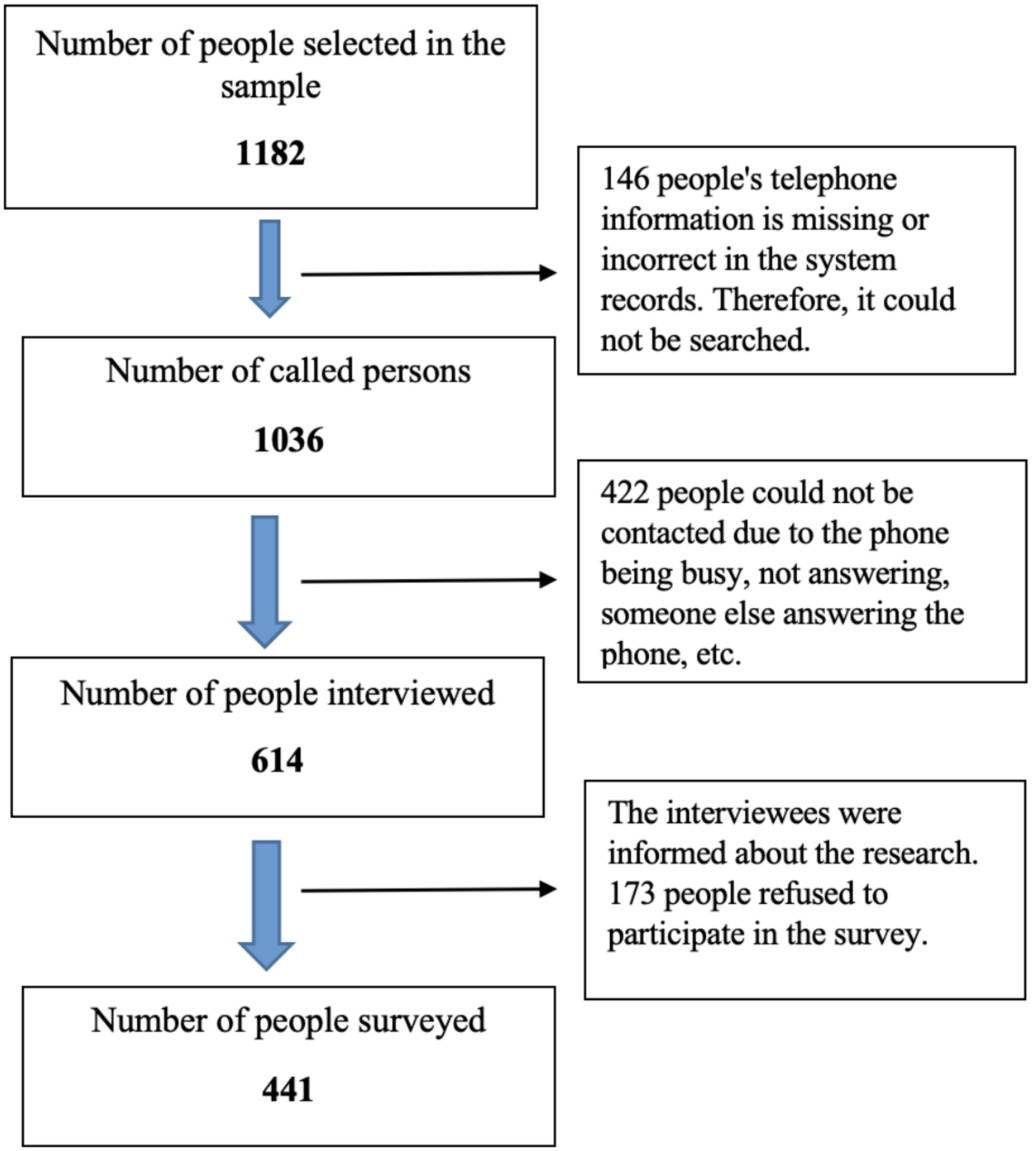
Flowchart of participant selection process.

The study population consisted of adults aged 18–65 years registered with a family physician in Sultanbeyli. Based on a known-population sample size calculation with a 95% confidence level and a 5% margin of error, the minimum required sample size was 394 participants. The sample frame for the study was created using current population lists of adults aged 18–65 registered at family health centers in the Sultanbeyli district. These lists were obtained through the family medicine information system, and each individual was assigned a unique identification number. To account for potential non-response associated with telephone-based data collection, three times the calculated sample size (1,182 individuals) was randomly selected from the sampling frame using a computer-based random number generation method. The selected individuals were contacted by telephone, and 441 participants who agreed to participate and completed the data collection process were included in the study. The participant selection process is presented in Figure 1. In this study, climate change–related health literacy was operationally defined as individuals’ ability to access, understand, evaluate, and apply information about the health impacts of climate change in their daily lives.

### Data collection instruments

Data were collected using a two-part questionnaire. The descriptive information form consisted of 10 items addressing occupational categories classified according to ILO standards and participants’ demographic and socioeconomic characteristics (23). Climate change–related health literacy was assessed using the 24-item Climate Change Health Literacy Scale (CCHLS) developed by Nayir et al. (24). The scale evaluates knowledge, attitudes, and behaviors related to the health impacts of climate change. Items are rated on a 5-point Likert scale, and the total scale score ranges from 24 to 120, with higher scores indicating greater knowledge, more positive attitudes, and more appropriate behaviors. Cut-off values for low, medium, or high levels have not been defined. In the present study, the scale demonstrated high internal consistency (Cronbach’s alpha = 0.94). The CCHLS comprises four subscales: health impact (11 items; score range: 11–55), follow-up (6 items; score range: 6–30), behavioral (2 items; score range: 2–10), and protection–support (5 items; score range: 5–25). However, the Behavioral subscale demonstrated low internal consistency (Cronbach’s alpha = 0.26) and was therefore excluded from the analyses. The dependent variables of the study were the total CCHLS score and subscale scores (health impact, follow-up, and protection–support). Independent variables included age, gender, education level, income status, occupational status, receipt of formal education on climate change, and following climate-related news. Domain scores were calculated by summing item responses within each subscale, and higher scores indicated higher levels of knowledge, more positive attitudes, and more appropriate behaviors related to the health impacts of climate change.

### Data collection procedure

Data were collected through telephone interviews conducted by trained personnel from the District Health Directorate. Participants were informed about the purpose and voluntary nature of the study, and verbal informed consent was obtained prior to participation. Interviews lasted approximately 8–10 min. All data were anonymized and handled in accordance with data protection regulations. Participants were included if they were between 18 and 65 years of age, registered with a family physician within the Sultanbeyli district, able to speak and understand Turkish, reachable by telephone and agreed to participate in the interview, and volunteered to participate by providing verbal consent. Individuals who were younger than 18 years or older than 65 years, or who could not be reached by telephone (e.g., due to an invalid or inaccessible phone number), were excluded from the study.

### Statistical analysis

Data were analyzed using SPSS version 26. Normality was assessed using Q–Q plots and skewness and kurtosis values within ±2 (25). Independent samples *t*-tests and one-way ANOVA were used to compare mean scores. *Post-hoc* analyses were conducted where appropriate. Statistical significance was set at *p* < 0.05. Subscale analyses were performed according to the, Health impact, Follow up, Protection-Support as defined in the original scale form.

## Results

The study included 441 individuals aged 18–65 years, with a mean age of 38.66 ± 12.11 years. More than half of the participants were female, had completed primary education, reported income equal to expenses, and were not employed. Most participants had not received formal education on climate change and reported social media as their primary source of climate-related information. Descriptive characteristics of the study population are presented in Table 1. Comparisons of CCHLS total scores across participant characteristics revealed statistically significant differences according to gender, educational level, receipt of formal climate change education, following climate change–related news, and self-reported knowledge of the health impacts of climate change (*p* < 0.05). Higher CCHLS total scores were observed among women, university graduates, individuals who had received formal climate change education, those who followed climate-related news, and participants reporting sufficient knowledge of climate-related health impacts. No statistically significant differences were found according to occupational group or income status (*p* > 0.05) (Table 1).

**Table 1:** CCHLS scores according to some characteristics of participants (*N* = 441).

Variables	Number	Mean ± SD	*t*/*F*	*p*	*Post-hoc*
Gender					
Female	235	95.95 ± 12.07	2.502	**0.013**	**—**
Male	206	92.58 ± 15.72			
Educational status					
^a^Illiterate	20	87.35 ± 12.01	3.978	**0.002**	c > a^**^ c > b^**^
^b^Elementary school	186	92.48 ± 12.58			
High school	100	95.03 ± 15.39			
Associate degree	45	95.73 ± 15.31			
^c^Bachelor’s degree	82	99.18 ± 13.07			
Graduate	8	91.01 ± 19.19			
Occupational group					
Not working (housewife, student, retired)	195	94.71 ± 11.86	2.228	0.051	**—**
Professional (teacher, nurse, lawyer, engineer, architect, doctor)	70	98.32 ± 15.22			
Technical (technician, engineer)	22	92.72 ± 15.52			
Office worker (secretary)	29	94.58 ± 14.64			
Service and sales (sales consultant, cashier, cleaning staff, waiter, hairdresser)	99	92.56 ± 15.01			
Self-employed (entrepreneur)	26	89.38 ± 17.26			
Income					
Income is less than expenses	161	92.61 ± 13.13	2,409	0.091	—
Revenue equals expenses	228	95.05 ± 13.84			
Revenue exceeds expenses	52	96.90 ± 16.57			
Received education on climate change					
Yes	24	106.70 ± 10.28	4,540	**0.000**	—
No	417	93.67 ± 13.84			
Following climate change news					
^a^Not following	34	84.61 ± 14.71	10.465	**0.000**	b > a^***^ c > a^**^
^b^Follows on social media	287	95.84 ± 14.25			
^c^Follows via TV/Radio	120	93.64 ± 11.90			
Do you think you are aware of the health effects of climate change?					
^a^Yes	96	100.31 ± 13.76	33.782	**0.000**	a > c^***^ a > b^*^ b > c^***^
^b^Partially	236	95.83 ± 12.49			
^c^ No	109	86.01 ± 13.55			

SD, standard deviation; *post-hoc*, Tukey; *F*, one-way ANOVA; *t*, independent sample *t*.

Bold values: ^*^p < 0.05, ^**^p < 0.01, ^***^p < 0.001.

The mean CCHLS health impact subdimension score was 48.41 ± 8.67, the follow-up subdimension score was 16.12 ± 6.19, the behavior subscale score was 9.39 ± 1.24 and the protection-support subdimension score was 20.44 ± 4.43. The mean total CCHLS score was 94.38 ± 13.98 (Table 2).

**Table 2:** Participants’ CCHLS subdimensions scores, min–max values, and Cronbach’s alpha coefficients (*N* = 441).

Subdimensions	Mean ± SD	Min–Max	Cronbach’s alpha
Health impact	48.41 ± 8.67	11–55	0.89
Follow-up	16.12 ± 6.19	6–30	0.76
Behavioral	9.39 ± 1.24	2–10	0.26
Protection-support	20.44 ± 4.43	5–25	0.71
Total	94.38 ± 13.98	24–120	0.84

Min, minimum, Max, maximum.

Subscale analyses showed a statistically significant difference in health impact scores according to gender, with women scoring higher than men (*p* < 0.01). No statistically significant differences in health impact scores were observed according to educational level, occupational group, income status, receipt of climate change education, or following climate-related news (*p* > 0.05) (Table 3).

**Table 3:** CCHLS—health impact dimension scores according to some characteristics of participants (*N* = 441).

Variables	Number	Mean ± SD	*t*/*F*	*p*
Gender				
Female	235	49.71 ± 6.75	3.403	0.001*
Male	206	46.92 ± 10.25		
Educational status				
^a^Illiterate	20	46.90 ± 7.81	1.133	0.342
^b^Primary education	186	48.01 ± 8.85		
High school	100	48.75 ± 8.29		
Associate degree	45	47.84 ± 9.24		
^c^Bachelor’s degree	82	49.97 ± 7.79		
Post graduate	8	44.50 ± 14.65		
Occupational group				
Not working (housewife, student, retired)	195	49.15 ± 6.74	1.440	0.209
Professional (teacher, nurse, lawyer, engineer, architect, doctor)	70	48.94 ± 9.17		
Technical (technician, engineer)	22	48.77 ± 6.87		
Office worker (secretary)	29	49.34 ± 7.24		
Service and sales (sales consultant, cashier, cleaning staff, waiter, hairdresser)	99	46.68 ± 10.99		
Self-employed (entrepreneur)	26	46.57 ± 12.02		
Income				
Income is less than expenses	161	47.96 ± 9.22	0.365	0.694
Revenue equals expenses	228	48.73 ± 8.24		
Revenue exceeds expenses	52	48.36 ± 8.83		
Received education on climate change				
Yes	24	51.16 ± 7.17	1.911	0.067
No	417	48.25 ± 8.73		
Following climate change news				
^a^Not following	34	45.55 ± 9.33	2.914	0.055
^b^Follows on social media	287	49.02 ± 8.32		
^c^Follows via TV/Radio	120	47.75 ± 9.14		

SD, standard deviation; *post-hoc*, Tukey; *F*, one-way ANOVA; *t*, independent samples *t*.

Bold values: ^*^p < 0.05.

Statistically significant differences were found between follow-up dimension scores and the variables educational status, occupational group, income, receiving education on climate change, and following climate change news (*p* < 0.001). Accordingly, participants with high school and university degrees, those in professional occupational groups, those with incomes equal to or higher than their expenses, those who had received education on climate change, and those who followed climate change news had higher follow-up scores than others. *Post-hoc* analysis was performed for characteristics with more than two categorical variables (Table 4).

**Table 4:** CCHLS—follow-up dimension scores according to some characteristics of participants (*N* = 441).

Variables	Number	Mean ± SD	*t*/*F*	*p*	*Post-hoc*
Gender					
Female	235	15.60 ± 6.22	−1.923	0.055	—
Male	206	16.73 ± 6.12			
Educational status					
^a^Illiterate	20	11.01 ± 4.55	13.397	**0.000**	c > a^**^ d > a^***^ e > a^***^
^b^Elementary education	186	14.37 ± 5.83			
^c^High school	100	16.53 ± 5.71			
^d^Associate’s degree	45	18.66 ± 5.95			
^e^Bachelor’s degree	82	19.26 ± 5.96			
^f^Graduate	8	18.37 ± 6.16			
Occupational group					
^a^Not working (housewife, student, retired)	195	14.82 ± 6.01	6.502	**0.000**	b > a^***^ b > c^*^ b > d^*^
^b^Professional (teacher, nurse, lawyer, engineer, architect, doctor)	70	19.48 ± 5.92			
Technical (technician, engineer)	22	16.50 ± 6.27			
Office worker (secretary)	29	16.75 ± 5.28			
^c^Service and sales (sales consultant, cashier, cleaning staff, waiter, hairdresser)	99	16.36 ± 6.23			
^d^Self-employed (entrepreneur)	26	14.96 ± 5.92			
Income					
^a^Income is less than expenses	161	14.85 ± 6.07	8.341	**0.000**	b > a^*^ c > a^***^
^b^Revenue equals expense	228	16.44 ± 6.04			
^c^Income exceeds expenses	52	18.67 ± 6.40			
Receiving education on climate change					
Yes	24	23.83 ± 4.67	8.157	**0.000**	—
No	417	15.68 ± 5.98			
Following climate change news					
^a^Not following	34	11.58 ± 4.97	14.149	**0.000**	b > a^***^ c > a^**^ b > c^*^
^b^Follows on social media	287	17.03 ± 6.31			
^c^Follows via TV/Radio	120	15.25 ± 5.54			

SD, standard deviation; *post-hoc*, Tukey, *F*, one-way ANOVA; *t*, independent samples *t*.

Bold values: ^*^p < 0.05, ^**^p < 0.01, ^***^p < 0.001.

Statistically significant differences were found between participants’ Protection-Support dimension scores and gender, occupational group, and following climate change news (*p* < 0.05). Women, not working participants, and those who followed climate change news on TV/Radio had higher scores than others. No statistically significant differences were found between Protection-Support dimension scores and the variables Educational status, Income, and Receiving education on climate change (*p* > 0.05) (Table 5).

**Table 5:** CCHLS—protection-support dimension scores according to some characteristics of the participants (*N* = 441).

Variables	Number	Mean ± SD	*t*/*F*	*p*	*Post-hoc*
Gender					
Female	235	21.17 ± 3.71	3.734	**0.000**	—
Male	206	19.61 ± 5.02			
Educational status					
^a^Illiterate	20	20.10 ± 3.74	0.630	0.677	—
^b^Elementary education	186	20.77 ± 4.13			
High school	100	20.37 ± 4.99			
Associate degree	45	19.97 ± 4.77			
^c^Bachelor’s degree	82	20.28 ± 4.35			
Post graduate	8	18.62 ± 4.80			
Occupational group					
^a^Not working (housewife, student, retired)	195	21.23 ± 3.67	3.297	**0.006**	a > b^*^
Professional (teacher, nurse, lawyer, engineer, architect, doctor)	70	20.22 ± 4.48			
^b^Technical (technician, engineer)	22	18.36 ± 6.04			
Office worker (secretary)	29	19.73 ± 5.57			
Service and sales (sales consultant, cashier, cleaning staff, waiter, hairdresser)	99	20.12 ± 4.43			
Self-employed (entrepreneur)	26	18.76 ± 5.57			
Income					
Income is less than expenses	161	20.51 ± 4.15	0.031	0.936	—
Income equals expenses	228	20.39 ± 4.56			
Income exceeds expenses	52	20.44 ± 4.77			
Receiving education on climate change					
Yes	24	22.12 ± 2.84	1.916	0.056	—
No	417	20.34 ± 4.49			
Following climate change news					
^a^Not following	34	18.70 ± 5.10	3.828	**0.022**	b > a^*^
Follows on social media	287	20.39 ± 4.64			
^b^Follows via TV/Radio	120	21.05 ± 3.52			

SD, standard deviation; *post-hoc*, Tukey; *F*, one-way ANOVA; *t*, independent samples *t*.

Bold values: ^*^p < 0.05, ^**^p < 0.01, ^***^p < 0.001.

Overall, the findings of the study demonstrate that climate change–related health literacy levels vary significantly across sociodemographic and behavioral characteristics. Higher health literacy scores were consistently observed among women, individuals with higher educational attainment, those who had received formal education on climate change, and participants who followed climate-related news. In contrast, occupational status and income level showed limited or inconsistent associations with health literacy outcomes. These results highlight the differential distribution of climate change–related health literacy in the population and underscore the importance of targeted educational and communication strategies, particularly for groups with lower literacy levels.

## Discussion

The mean CCHLS health impact subdimension score was 48.41 ± 8.67, the follow-up subdimension score was 16.12 ± 6.19, the behavior subscale score was 9.39 ± 1.24, and the protection-support subdimension score was 20.44 ± 4.43. The mean total CCHLS score was 94.38 ± 13.98. CCHLS subdimension scores indicated that participants were relatively aware of health impacts, yet follow-up and protection-support behaviors were inconsistent. This finding aligns with the frequently reported knowledge–behavior gap in the literature, showing that awareness of health impacts alone does not guarantee appropriate monitoring or protective actions. In particular, the low follow-up scores suggest that participants were limited in applying what they had learned in daily life, while the moderate protection-support scores indicate that such behaviors were occasionally performed but not consistently maintained. These findings highlight the need for climate-related health literacy interventions that go beyond providing information to include strategies that actively support follow-up and protective behaviors.

Previous studies have reported that awareness of climate change does not automatically translate into health-protective behaviors, particularly among vulnerable populations (3, 11). Evidence from Turkey also supports this pattern, as public concern about climate change appears relatively high, while climate-related health literacy remains limited due to insufficient technical and health-focused knowledge (19). In the present study, CCHLS subdimension scores indicated that participants were relatively knowledgeable about health impacts, yet follow-up and protection-support behaviors were inconsistent. Similar findings have been reported in Egypt, Italy, and Turkey, where high awareness and concern did not always lead to effective action (26–28). These results underscore the need for interventions that not only provide information but also actively support follow-up and protective behaviors, highlighting that climate-related health literacy functions not only as an individual cognitive skill but also as a determinant of community adaptive capacity. Climate change related health literacy functions not only as an individual cognitive skill but also as a determinant of community adaptive capacity. In socioeconomically disadvantaged settings characterized by limited educational opportunities and increased exposure to environmental stressors, insufficient climate-health literacy may reinforce existing health inequalities and constrain effective adaptation to climate-related health risks. The observed imbalance between knowledge and behavior highlights the need for interventions that go beyond information provision and actively support behavior change at the community level.

Our finding that higher total CCHLS scores were observed among women, university graduates, individuals who had received formal climate change education, those who followed climate related news, and participants reporting sufficient knowledge of climate related health impacts. Women scored higher than men on the health impact and protection-support subscales. In contrast, no significant differences were observed according to income level or occupational status, suggesting that access to reliable information and education may be more influential than economic factors alone in shaping climate-health literacy. Previous studies suggest that women often demonstrate higher levels of environmental and health related literacy, potentially due to greater engagement with health information and educational content, and this has been echoed in research exploring women’s environmental and climate literacy and empowerment in climate action contexts (14, 29–32). In addition, systematic reviews have shown that women tend to demonstrate slightly higher levels of general health literacy compared to men (33). However, there is also a study that found women’s knowledge level regarding the effects of climate change on health to be insufficient or moderate (34). In this study, the higher total CCR-HLS scores of women can be explained by the inclusion of attitude and behavior dimensions in addition to knowledge dimensions in the scale, and by women being more active in accessing and applying health information. In line with this evidence, the higher scores observed among women in the present study suggest the need for gender-sensitive climate–health communication strategies, particularly interventions tailored to improve engagement and behavioral responses among men.

Similarly, the association between educational attainment and climate-health literacy underscores education as a key determinant of health literacy and adaptive capacity (13, 24). These findings suggest that women and more educated individuals may serve as important facilitators of climate-aware health behaviors within their communities.

Subscale analyses revealed that women scored significantly higher than men on the health impact subdimension, suggesting greater awareness or sensitivity among female participants regarding climate-related health risks. No significant differences were observed based on education, occupation, income, receipt of climate change education, or following climate-related news, indicating that these factors alone may not strongly influence perceived health impacts.

Follow-up scores differed significantly according to education, occupation, income, receipt of climate change education, and following climate-related news, with higher scores observed among participants with high school or university degrees, professional occupations, sufficient income, prior climate education, and news engagement. These findings suggest that follow-up behaviors are influenced not only by awareness but also by access to education, resources, and opportunities for informed engagement.

Protection-Support scores differed significantly by gender, occupational status, and following climate change news with higher scores observed among women, non-working participants, and those who followed news via TV or radio. No significant differences were found based on education, income, or receipt of climate change education, suggesting that protective and supportive behaviors are influenced more by gender, time availability, and media engagement than by formal education or income. The World Health Organization’s report highlights that the health impacts of climate change differ by gender, and that these differences are significant for adaptation and protection strategies (32).

This study highlights that while participants demonstrated awareness of the health impacts of climate change, follow-up and protection-support behaviors were inconsistent. Higher climate-health literacy was observed among women, university graduates, individuals who had received formal climate education, and those regularly following climate-related news, emphasizing the role of gender, education, and access to reliable information in shaping adaptive capacity. The lack of significant differences by income or occupation suggests that education and information access are more influential determinants than economic factors alone. The majority of participants had not received formal climate education, and reliance on social media as a primary information source presents both opportunities and challenges for effective risk perception and protective behaviors. These findings underscore the critical role of public health nurses in strengthening community-based education, correcting misinformation, and promoting protective behaviors. Gender-sensitive approaches and strategies that actively support follow-up and protective actions are essential to translate awareness into sustained action and mitigate the health impacts of climate change.

A notable finding of this study is that the vast majority of participants had not received formal education on climate change, indicating a substantial gap in community-level climate-health capacity. Although participants who regularly followed climate-related information demonstrated higher literacy scores, relying on social media as the primary source presents both opportunities and challenges. While digital platforms facilitate rapid information dissemination, they also increase exposure to misinformation, which may negatively affect risk perception and protective behaviors (11). In this context, climate change–related health literacy should be considered a public health priority aimed at strengthening community adaptive capacity and reducing vulnerability.

Public health nurses are well positioned to address knowledge gaps due to their sustained engagement with individuals and communities. Through education, advocacy, and dissemination of evidence-based information, they can enhance critical appraisal skills, correct misinformation, and promote protective behaviors related to climate-related health risks. Consistent with World Health Organization recommendations, community empowerment through education represents a key strategy for mitigating the health impacts of climate change (3). In this context, public health nurses play a critical role by providing community-based education, individual risk assessments and counseling for vulnerable groups, and promoting protective behaviors and awareness of water and food safety. Measurable outcomes include increased health literacy, adoption of adaptive behaviors, reduced exposure to environmental risks, and strengthened self-management skills (35, 36). The finding that only 21% of participants perceived themselves as knowledgeable about the health impacts of climate change underscores the urgent need for targeted, nursing-led educational interventions in socioeconomically disadvantaged urban settings.

## Conclusion

In conclusion, this study demonstrates that while participants showed general awareness of the health impacts of climate change, follow-up and protection-support behaviors were inconsistent, reflecting a persistent knowledge–behavior gap. Higher climate-health literacy was observed among women, university graduates, individuals who had received formal climate change education, those who regularly followed climate-related news, and participants reporting sufficient knowledge of climate-related health impacts, highlighting the influence of gender, education, and access to information on community adaptive capacity. No significant differences were found according to occupational group or income status, suggesting that access to education and reliable information may be more critical than economic or occupational factors in shaping climate-health literacy. The majority of participants had not received formal education on climate change, and reliance on social media as a primary information source presents both opportunities and challenges for effective risk perception and protective behaviors. These findings emphasize the critical role of public health nurses in enhancing climate-health literacy through targeted, community-based education and advocacy. Interventions should extend beyond providing information to actively support follow-up and protective behaviors, incorporate gender-sensitive strategies, and strengthen community adaptive capacity, particularly in socioeconomically disadvantaged settings.

### Strengths and limitations

This study has several strengths. The fact that it was conducted in a socioeconomically disadvantaged urban area and focused on an under-researched and vulnerable population is a significant advantage. The relatively large sample size (*N* = 441) and the assessment of health literacy regarding climate change using a valid and reliable measurement tool enhance the reliability of the findings. However, there are also some limitations. Due to the cross-sectional design, causal relationships between variables cannot be determined. Macro-level determinants (industrial activities, energy consumption, the role of institutional actors) were excluded; examining these factors would require different datasets and methods. Since the study focuses solely on the Marmara Region and Istanbul, generalizations to the whole of Turkey are limited. Collecting data via telephone interviews may have limited the depth of responses and potentially created a bias toward non-response. Additionally, the fact that the study was conducted in a single district of Istanbul limits the generalizability of the findings to other regions. Finally, the Behavior subscale, consisting of two items, was excluded from the analyses due to its low internal consistency (Cronbach’s *α* = 0.26).

## Data Availability

The raw data supporting the conclusions of this article will be made available by the authors, without undue reservation.
